# Exceptional Long-Term Survival of a Patient With Hepatoid Adenocarcinoma of the Colon and the Treatment Strategy: A Case Report

**DOI:** 10.7759/cureus.55620

**Published:** 2024-03-06

**Authors:** Honghua Jiang, Guoping Fang, Jiwei Zhang

**Affiliations:** 1 Department of Colorectal Surgery, Xinhua Hospital Affiliated to Shanghai Jiao Tong University School of Medicine, Shanghai, CHN; 2 Department of Interventional Radiology, Xinhua Hospital Affiliated to Shanghai Jiao Tong University School of Medicine, Shanghai, CHN

**Keywords:** alpha-fetoprotein, case report, liver metastasis, colorectal, hepatoid adenocarcinoma

## Abstract

Hepatoid adenocarcinoma (HAC) of the colon is a rare type of tumor with hepatocellular differentiation. HAC often produces alpha-fetoprotein (AFP) and metastasizes to lymph nodes and the liver. HAC is usually aggressive with a poor prognosis and has a propensity for intravascular growth and frequent distant metastasis. Because the biology of HAC is not fully understood, there are very limited therapeutic options known to reduce recurrence and improve survival. In addition, because HAC is so rare, it is difficult to acquire data from large randomized clinical trials to guide practice; therefore, case reports can provide valuable information for the treatment of HAC. In this report, we present a case of a 30-year-old male patient with HAC with high AFP levels and liver metastases. The patient underwent hepatic arterial infusion chemotherapy (HAIC) with doxorubicin/oxaliplatin to treat the liver metastasis, and three weeks later, he received radical sigmoid and rectal resection, left liver resection, and ileostomy. Then, the patient received eight cycles of chemotherapy with epirubicin plus folinic acid, fluorouracil, and oxaliplatin (FOLFOX) every three weeks, followed by maintained therapy with capecitabine for 2.5 years without relapse. This case report indicates that, although HAC is usually an aggressive disease with frequent distant metastasis, patients with HAC may still have a good prognosis if treated with appropriate strategy.

## Introduction

Hepatoid adenocarcinoma (HAC) is a rare variant of adenocarcinoma. HAC was first reported as an α-fetoprotein (AFP)-producing tumor by Bourreille et al. in 1970, and the term was coined by Ishikura et al. [1,2]. AFP is classified as a member of an albuminoid gene family, which consists of four members to date: albumin (ALB), vitamin D-binding (Gc) protein (DBP), AFP, and alpha-ALB (aALB), termed afamin in humans [3]. The AFP structure is similar to that of ALB. However, the functions of which are different [4]. ALB maintains stable plasma osmolality and delivers nutrients. AFP delivers nutrients, suppresses immunity, and stimulates the growth of cancer cells. When the serum concentration of AFP is greater than 50 ng/mL in adult blood, it stimulates tissue regeneration or hematopoiesis, and it is also used by cancer cells to provide nutrients and stimulate growth [5]. High expression of AFPR (AFP receptor) has been observed in the membrane of HCC cells [6], and AFP binds with AFPR, which increases the concentrations of cAMP and Ca2+ in the cytoplasm and promotes the expression of some oncogenes [7]. Secreted AFP has many functions, such as immunosuppression, and it regulates the malignant behaviors of cancer cells through mediation by AFPR. AFP binds to phosphatase and tensin homolog (PTEN) in the cytoplasm and activates PI3K signaling pathways, thus stimulating the growth of many malignant cells [8].

HAC is most frequently identified in the stomach [9] but is also found in the ovary, lung [10], pancreas [11], gallbladder, cervix, and thymus [12]. HAC is a very aggressive neoplasm with an unfavorable prognosis and a high rate of metastases at the time of diagnosis, with the liver and lymph nodes being the most common metastatic sites [13]. It is extremely rare that AFP is produced in colorectal cancer [14]. According to a study by Hu et al. [15], until 2018, only 17 cases of HAC of colorectal cancer had been reported in the English literature through a PubMed search. Adding their case, there were 12 patients with liver metastases and 11 patients with lymph node metastases [15]. Despite aggressive treatment with radical surgery, followed by adjuvant chemotherapy, 12 patients died within the first 12 months, and the median overall survival was eight months. currently, due to the rare case, the treatment of metastatic HAC (mHAC) remains unclear. In this report, we present a case of HAC with multiple metastasis foci in the liver and lots of lymph nodes in the sigmoid and rectal mesentery. After hepatic arterial infusion chemotherapy (HAIC) and surgery, followed by chemotherapies, no recurrence occurs within the four-year follow-up period.

## Case presentation

**Figure 1:**
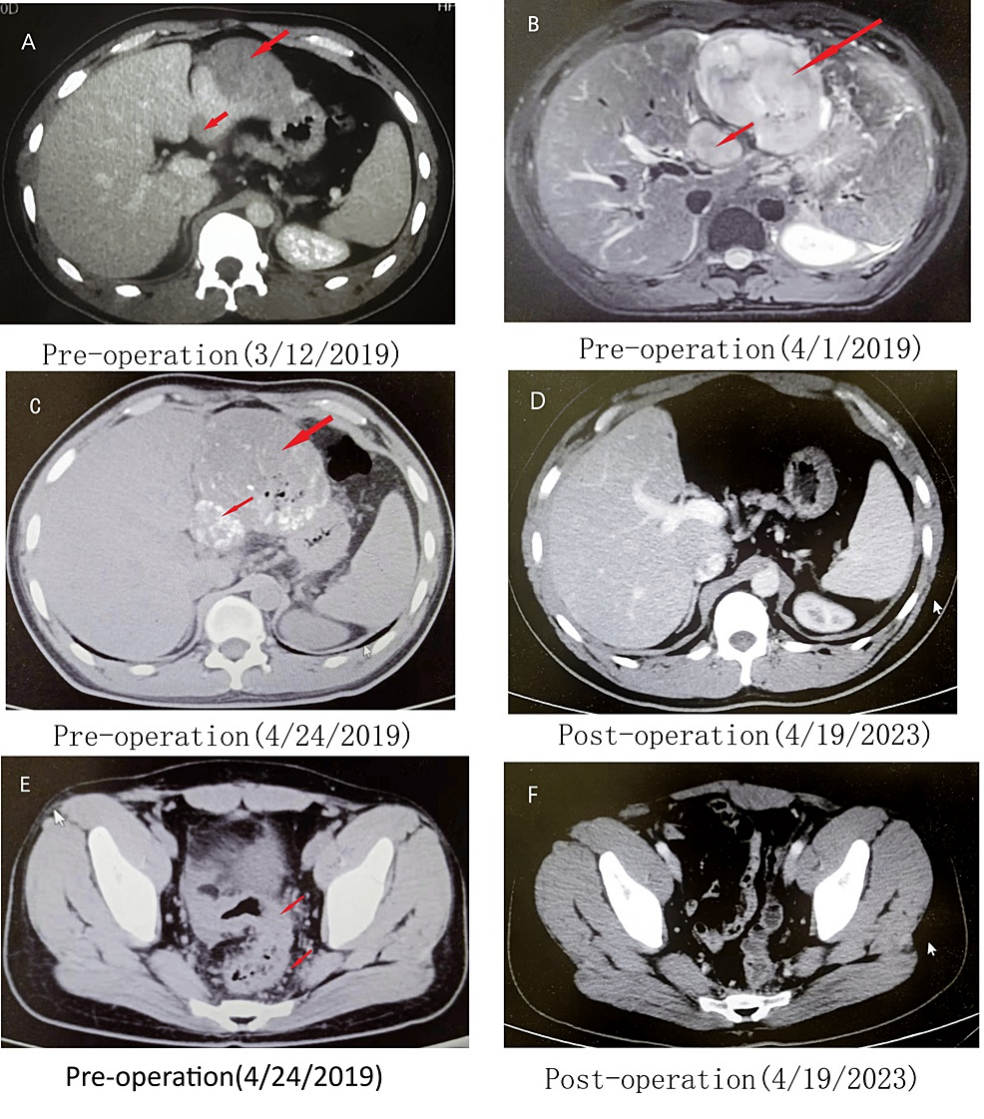
CT and MRI images A: Two metastasis foci in the left liver lobe in the CT image. B: The metastasis foci in the MRI image three weeks later. C: Post-HAIC treatment, metastasis foci in the CT scan before operation. D: The liver CT scan at four years post operation. E: There were lots of lymph nodes in the rectal mesenteric in the pelvic CT pre operation. F: The pelvic CT scan at four years post operation.

**Figure 2:**
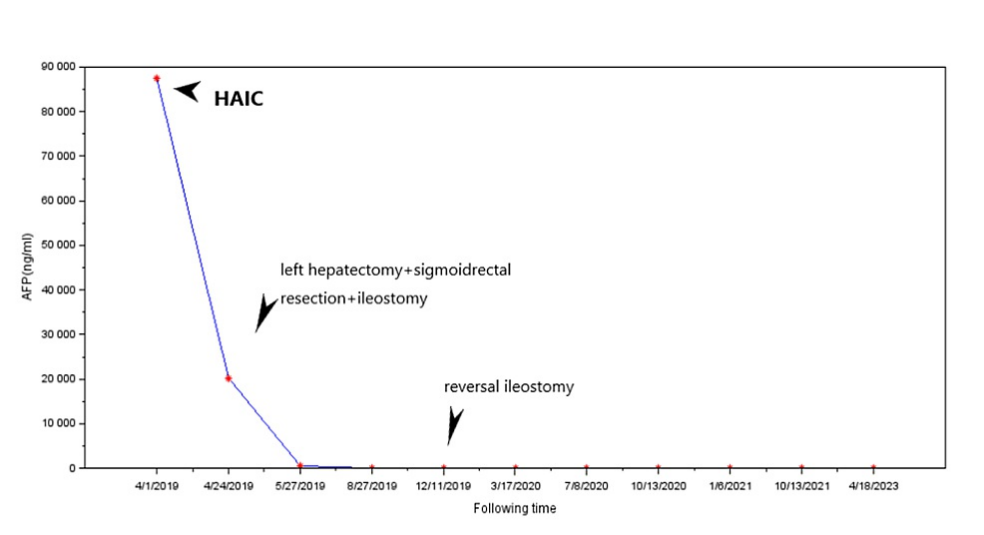
Level of serum alpha-fetoprotein (AFP) After the hepatic arterial infusion chemotherapy (HAIC), the serum AFP dropped from 87,438 ng/mL (4/1/2019) to 20,261 ng/mL (4/24/2019). After left hepatectomy and radical sigmoid as well as rectum resection, the serum AFP dropped to 542 ng/mL and then to 4.29 ng/mL (8/29/2019) after four cycles of chemotherapy with FOLFOX+epirubicin.

A male 30-year-old patient had a colonoscopy that showed a mass in the sigmoid in another hospital due to hematochezia for more than two months. CT showed multiple metastasis foci in the left liver, with the diameter of the largest mass of 5 cm (Figure 1A). The patient was admitted to Xinhua Hospital three weeks later, and the MRI showed that the mass had increased to 10 cm (Figure 1B), and there was little gap between the metastasis foci and hepatic portal vein. The serum AFP was 87,438 ng/mL (Figure 2). The patient received HAIC with oxaliplatin and doxorubicin. Three weeks later, the preoperative laboratory testing showed that the AFP dropped to 20,261 ng/mL, and the high-level AFP stimulated the growth of HAC, CT images showed that the liver metastasis foci still growing (Figure* *1C), the pelvic CT showed sigmoid colon mass infiltrated to the rectum at the level of reflex of the peritoneum, and the sigmoid colon and rectal mesentery showed numerous lymph nodes (Figure 1E).

For the treatment, left hepatectomy and radical sigmoid as well as rectum resection were performed. During the operation, there was a 10 cm mass in the left hepatic lobe and one mass with a diameter of about 4 cm in four segments. There were lots of lymph nodes in the sigmoid and rectal mesentery. After removing the left liver, the splenic flexure of the colon was freed, and the sigmoid colon and rectum were resected according to total mesorectal excision (TME). The anastomosis between the descending colon and rectum was made at the pelvic floor level, and ileostomy was also performed. The postoperative recovery was stable. After eight cycles of chemotherapy with folinic acid, fluorouracil, and oxaliplatin (FOLFOX) plus epirubicin, the ileostomy was closed. Oral capecitabine was given as maintenance therapy for 2.5 years. The four-year post-operation follow-up showed no signs of recurrence (Figures 1D-1F).

This patient presented with a very high serum AFP level. The serum AFP before chemotherapy was 87,438 ng/mL (Figure 2). However, three weeks later, the patient received HAIC with oxaliplatin and doxorubicin, and the preoperative laboratory testing showed that the AFP dropped to 20,261 ng/mL (Figure 2). After the one-stage radical resection of the colorectal lesion and liver metastasis foci, the serum AFP level further dropped to 542 ng/mL (Figure 2).

## Discussion

Here, we presented an aggressive HAC case with a very high AFP level, and the size of liver metastasis foci was doubled within three weeks. Although the serum AFP levels dropped from 87,438 ng/mL to 20,261 ng/mL after the HAIC, an increase in the liver metastasis size was still observed, though the growth velocity of metastasis loci was reduced. After the one-stage radical resection of the colorectal lesion and liver metastasis foci, the serum AFP level dropped to 542 ng/mL. After eight cycles of chemotherapy with FOLFOX and epirubicin, followed by capecitabine maintenance treatment for 2.5 years, this patient achieved a very long-time recurrence-free survival (within four years).

What we learned from this case for HAC treatment is that radical resection of the primary and metastasis foci is very important to remove the primary and metastasis lesions. If this is not feasible, the debulking operation will be the second option [16], and the last may be the stereotactic body radiation therapy (SBRT) [17]. If none of the above options are possible, the prognosis is very poor [18]. The second important approach to treating HAC is to choose the effective chemotherapy. In this case, doxorubicin plus oxaliplatin-based HAIC dramatically reduced the AFP level. In previous reports, most of the successful treatments of HAC were also platinum-based chemotherapy [19]. The adjuvant chemotherapy with FOLFOX plus epirubicin and capecitabine maintenance therapy works very well to prevent the recurrence. HAC is very rich in angiogenesis compared to other cancers, and most cancer cells produce vascular endothelial growth factor (VEGF), which is involved in the mitosis of endothelial cells in vitro and is a factor of angiogenesis in vivo. Ramucirumab inhibits VEGF-A, VEGF-C, and VEGF-D by blocking VEGF-R2, and the same targeted drug may be effective for AFP-producing gastric cancer [9]. For HER2-positive gastric hepatoid adenocarcinoma, cisplatin/capecitabine plus trastuzumab was reported effective [20]. The target therapeutic drugs may play more important roles in treating HAC in the future.

## Conclusions

This case is an advanced colorectal HAC with multiple liver metastasis loci and very high AFP levels, which usually have a very poor prognosis. However, after appropriate treatment and care, the patient still achieved long-term recurrence-free survival. The case presented in this report also suggests that effective chemotherapy and radical resection played vital roles in HAC treatment. Although patient responses might be different to the treatments in different HAC cases, the strategies and treatment regimens used in the presented case might provide useful information for the treatment of other HACs.
